# Supplementary material for: Competition between lysogenic and sensitive bacteria is determined by the fitness costs of the different emerging phage-resistance strategies
Source: eLife. 2023 Mar 28;12:e83479. doi: 10.7554/eLife.83479 (PMC10076033; doi:10.7554/eLife.83479)
Supplement: Supplementary file 1. — The genome was analyzed with PHASTER (Arndt et al., 2016) in April 2021. [file elife-83479-supp1.docx]

| **Phage #** | **Length (kb)** | **Classification** | **Coding proteins** | **Comment** |
| --- | --- | --- | --- | --- |
| 1 | 108.9 | intact | integrase, recombinase, terminase, capsid, tail, lysin | Can lysogenize BJ1 (34) |
| 2 | 17.2 | incomplete | integrase, head, tail | P2 remnant? |
| 3 | 52.8 | intact | tail, head, terminase, transposase, lysis, integrase | Can lysogenize BJ1 (34) |
| 4 | 45.5 | intact | tail, capsid, terminase, head |  |
| 5 | 46.4 | intact | integrase, tail, terminase, head, protease, capsid |  |
| 6 | 47.6 | questionable | integrase, recombinase, tail, terminase, head, coat |  |
| 7 | 12.9 | incomplete | tail genes | Most likely not functional. No packaging genes |
| 8 | 12.7 | incomplete | tail genes | Most likely not functional. No packaging genes |
| 9 | 8.7 | incomplete | recombinase, transposase | Most likely not functional. |
| 10 | 10.2 | incomplete | transposase, tail, portal, terminase | Most likely not functional |
