# Supplementary material for: Competition between lysogenic and sensitive bacteria is determined by the fitness costs of the different emerging phage-resistance strategies
Source: eLife. 2023 Mar 28;12:e83479. doi: 10.7554/eLife.83479 (PMC10076033; doi:10.7554/eLife.83479)
Supplement: Supplementary file 2. — ND; none detected. On the day in which at least 50% of the clones of each independently evolving population were non-capsulated, two of such clones were isolated (# Clone), the wcaJ gene amplified by PCR and sequenced by Sanger. For BJ1, this was repeated twice independently (# Seq) (N=36 for BJ1 and N=18 for ST14). Independently evolving populations (Pop) are identified with a number from 1 to 3. Sequencing of the ancestor revealed that no mutations were present in the wcaJ genes. [file elife-83479-supp2.docx]

| Strain | # Seq | # Clone | Pop | Condition | Day | Variation | Detail | Position | Mutation  type | Aminoacid Change |
| --- | --- | --- | --- | --- | --- | --- | --- | --- | --- | --- |
| BJ1 | I | 1 | 1 | + Citrate | 8 | Insertion | +1 | 310 | frameshift | +G |
| BJ1 | I | 2 | 1 | + Citrate | 8 | SNP | T>A | 1195 | non-synonymous | Y399N |
| BJ1 | II | 1 | 1 | + Citrate | 8 | Deletion | -1 | 127 | frameshift | -T |
| BJ1 | II | 2 | 1 | + Citrate | 8 | SNP | A>C | 1196 | non-synonymous | Y399S |
| BJ1 | I | 1 | 1 | LB | 6 | SNP | A>C | 1196 | non-synonymous | Y399S |
| BJ1 | I | 2 | 1 | LB | 6 | SNP | C>T | 937 | Stop | Q312* |
| BJ1 | II | 1 | 1 | LB | 6 | SNP | A>C | 1196 | non-synonymous | Y399S |
| BJ1 | II | 2 | 1 | LB | 6 | ND | ND | ND | ND | ND |
| BJ1 | I | 1 | 1 | + MMC | 4 | SNP | A>C | 1196 | non-synonymous | Y399S |
| BJ1 | I | 2 | 1 | + MMC | 4 | SNP | G>A | 698 | non-synonymous | C233Y |
| BJ1 | II | 1 | 1 | +MMC | 4 | Deletion | -1 | 92 | frameshift | -T |
| BJ1 | II | 2 | 1 | + MMC | 4 | SNP | G>A | 698 | non-synonymous | C233Y |
| BJ1 | I | 1 | 2 | + Citrate | 8 | SNP | G>A | 698 | non-synonymous | C233Y |
| BJ1 | I | 2 | 2 | + Citrate | 8 | Insertion | +1 | 310 | frameshift | +G |
| BJ1 | II | 1 | 2 | + Citrate | 8 | SNP | A>C | 1196 | non-synonymous | Y399S |
| BJ1 | II | 2 | 2 | + Citrate | 8 | Insertion | 1 | 52 | frameshift | +T |
| BJ1 | I | 1 | 2 | LB | 6 | SNP | G>A | 698 | non-synonymous | C233Y |
| BJ1 | I | 2 | 2 | LB | 6 | SNP | G>A | 698 | non-synonymous | C233Y |
| BJ1 | II | 1 | 2 | LB | 6 | SNP | A>C | 1196 | non-synonymous | Y399S |
| BJ1 | II | 2 | 2 | LB | 6 | SNP | G>A | 698 | non-synonymous | C233Y |
| BJ1 | I | 1 | 2 | + MMC | 4 | SNP | G>A | 1090 | non-synonymous | E364K |
| BJ1 | I | 2 | 2 | + MMC | 4 | SNP | C>T | 937 | Stop | Q312* |
| BJ1 | II | 1 | 2 | + MMC | 4 | Insertion | +1 | 540 | frameshift | +T |
| BJ1 | II | 2 | 2 | + MMC | 4 | Insertion | +1 | 310 | frameshift | +G |
| BJ1 | I | 1 | 3 | + Citrate | 8 | SNP | A>C | 1196 | non-synonymous | Y399S |
| BJ1 | I | 2 | 3 | + Citrate | 8 | Insertion | 1 | 52 | frameshift | +T |
| BJ1 | II | 1 | 3 | + Citrate | 8 | SNP | A>C | 1196 | non-synonymous | Y399S |
| BJ1 | II | 2 | 3 | + Citrate | 8 | SNP | A>C | 1196 | non-synonymous | Y399S |
| BJ1 | I | 1 | 3 | LB | 6 | Insertion | 1 | 52 | frameshift | +T |
| BJ1 | I | 2 | 3 | LB | 6 | Insertion | 1 | 52 | frameshift | +T |
| BJ1 | II | 1 | 3 | LB | 6 | ND | ND | ND | ND | ND |
| BJ1 | II | 2 | 3 | LB | 6 | SNP | G>A | 1090 | non-synonymous | E364K |
| BJ1 | I | 1 | 3 | + MMC | 4 | Insertion | 1 | 52 | frameshift | +T |
| BJ1 | I | 2 | 3 | + MMC | 4 | SNP | A>C | 1196 | non-synonymous | Y399S |
| BJ1 | II | 1 | 3 | + MMC | 4 | SNP | A>C | 1196 | non-synonymous | Y399S |
| BJ1 | II | 2 | 3 | + MMC | 4 | Insertion | +1 | 310 | frameshift | +G |

| Strain | # Clone | Pop | Condition | Day | Variation | Detail | Position | Mutation  type | Aminoacid change |
| --- | --- | --- | --- | --- | --- | --- | --- | --- | --- |
| ST14 | 1 | 1 | + Citrate | 6 | Insertion | +1 | 540 | frameshift | +T |
| ST14 | 2 | 1 | + Citrate | 6 | Insertion | +1 | 540 | frameshift | +T |
| ST14 | 1 | 1 | LB | 6 | Deletion | -9 | 1273 | inframe deletion | -AAAATGGAA |
| ST14 | 2 | 1 | LB | 6 | Insertion | +1 | 540 | frameshift | +T |
| ST14 | 1 | 1 | + MMC | 2 | Insertion | +1 | 540 | frameshift | +T |
| ST14 | 2 | 1 | + MMC | 2 | Insertion | +1 | 540 | frameshift | +T |
| ST14 | 1 | 2 | + Citrate | 6 | Insertion | +1 | 540 | frameshift | +T |
| ST14 | 2 | 2 | + Citrate | 6 | Insertion | +1 | 540 | frameshift | +T |
| ST14 | 1 | 2 | LB | 6 | Insertion | +1 | 540 | frameshift | +T |
| ST14 | 2 | 2 | LB | 6 | SNP | C>A | 781 | non-synonymous | P261V |
| ST14 | 2 | 2 | LB | 6 | Insertion | +1 | 784 | frameshift | +T |
| ST14 | 2 | 2 | + MMC | 2 | Insertion | +1 | 540 | frameshift | +T |
| ST14 | 1 | 2 | + MMC | 2 | Insertion | +1 | 540 | frameshift | +T |
| ST14 | 2 | 3 | + Citrate | 6 | Insertion | +1 | 540 | frameshift | +T |
| ST14 | 1 | 3 | + Citrate | 6 | Insertion | +1 | 540 | frameshift | +T |
| ST14 | 2 | 3 | LB | 6 | Insertion | +1 | 540 | frameshift | +T |
| ST14 | 1 | 3 | LB | 6 | Insertion | +1 | 540 | frameshift | +T |
| ST14 | 2 | 3 | + MMC | 2 | Insertion | +1 | 540 | frameshift | +T |
| ST14 | 1 | 3 | + MMC | 2 | Insertion | +1 | 540 | frameshift | +T |
